# From Minor Monitoring to Major Insight: Predicting AF Development in the Congenital Heart Disease Population

**DOI:** 10.3390/jcdd13070293

**Published:** 2026-06-24

**Authors:** Can Zhang, Lixia Dai, Annemien E. van den Bosch, Vehpi Yildirim, Mathijs S. van Schie, Yannick J. H. J. Taverne, Natasja M. S. de Groot

**Affiliations:** 1 Department of Cardiology, Erasmus Medical Center, 3015 GD Rotterdam, The Netherlands; c.zhang.1@erasmusmc.nl (C.Z.); l.dai@erasmusmc.nl (L.D.); a.e.vandenbosch@erasmusmc.nl (A.E.v.d.B.); v.yildirim@erasmusmc.nl (V.Y.); m.vanschie@erasmusmc.nl (M.S.v.S.); 2 Department of Cardiology, Chongqing General Hospital, Chongqing 401147, China; 3 Department of Cardiothoracic Surgery, Erasmus Medical Center, 3015 GD Rotterdam, The Netherlands; y.j.h.j.taverne@erasmusmc.nl; 4 Department of Microelectronics, Signal Processing Systems, Faculty of Electrical Engineering, Mathematics and Computer Sciences, Delft University of Technology, 2628 CD Delft, The Netherlands

**Keywords:** congenital heart disease, atrial fibrillation, ectopic beats, PR interval, QTc interval, electrocardiography, Holter monitoring

## Abstract

Background: The prognostic value of electrocardiography (ECG)- and continuous rhythm monitoring (CRM)-derived markers for predicting atrial fibrillation (AF) onset and progression remains unclear in patients with congenital heart disease (CHD). Methods: We retrospectively analyzed 573 CHD patients who underwent 24 h Holter monitoring between 2003 and 2015. Baseline ECG and CRM parameters were assessed. Cox regression identified predictors of new-onset AF and AF progression, and interaction analyses explored effect modification by left atrial (LA) dilatation. Results: During 13 ± 5 years of follow-up, AF occurred in 107 patients (18.7%), of whom 32 (29.9%) progressed to persistent/permanent AF (PeAF). Patients with AF more frequently had prolonged PR and QTc intervals and higher atrial ectopy (AE) and ventricular ectopy burdens. Independent predictors of new-onset AF were older age, LA dilatation, higher AE burden, atrial tachycardia, and pacemaker implantation. AF progression was independently associated with older age, LA dilatation, higher AE burden, and prolonged PR interval. AE burden showed a stronger association with AF risk in patients without LA dilatation. Conclusions: In CHD patients, baseline ECG PR-intervals and CRM-derived AE burden independently predict AF onset and/or progression. These noninvasive markers may improve risk stratification and support earlier personalized rhythm management.

## 1. Introduction

Adults with congenital heart disease (CHD) face an increasing burden of atrial fibrillation (AF), characterized by earlier onset and greater lifetime risks than in individuals without CHD [1,2,3]. Development of AF in the CHD population is associated with serious long-term complications, including heart failure [4,5], cerebral vascular accident [6,7], and increased mortality [8,9]. Given the elevated risks and younger age at onset, early identification of individuals predisposed to AF is crucial, as early prediction enables early intervention, potentially preventing adverse outcomes.

Standard 12-lead electrocardiography (ECG) and continuous rhythm monitoring (CRM) are routinely employed in clinical follow-up of CHD patients. Among the CRM-derived parameters, atrial ectopy (AE) burden has emerged as a promising predictor of AF risk in CHD, especially when combined with clinical characteristics such as age, anatomical complexity, and left atrial (LA) dilatation [10]. In the general population, ECG-based conduction parameters, including PR interval, QRS duration, and corrected QT (QTc) interval, have also demonstrated predictive value for AF onset [11,12,13,14]. The prognostic relevance of these ECG parameters for AF onset in adults with CHD is unknown.

Progression from paroxysmal (PAF) to persistent or permanent AF (PeAF) poses an additional challenge. A previous study reported that 26% of CHD patients with PAF progressed to PeAF within just 3 years of initial diagnosis [15]. However, predictors of this rapid AF progression remain poorly defined. Whether baseline electrophysiological features derived from ECG recordings or CRM can identify patients at the highest risk for AF progression is an important but unresolved question. Therefore, we addressed these knowledge gaps by evaluating the prognostic significance of baseline ECG and CRM parameters for the prediction of AF new-onset and progression in a cohort of adult CHD patients during long-term follow-up.

## 2. Methods

### 2.1. Study Population

This retrospective study extends the Dysrhythmias in Patients with Congenital Heart Disease (DANARA) project. A total of 573 CHD patients evaluated at tertiary outpatient clinics who underwent a 24 h Holter registration between April 2003 and January 2015 were included. Follow-up in this study was defined as the time from the first CRM to the last visit or until April 2024, whichever occurred first. This study was approved by the Medical Ethics Committee of the Erasmus Medical Center, Rotterdam (MEC-2012–482). Informed consent of patients was not obligatory.

### 2.2. Data Assessment

Standard 12-lead ECGs and 24 h CRMs were independently reviewed by two experienced cardiologists. From every patient, the first available 24 h CRM was used to derive the number of AE, ventricular ectopy (VE), standard deviation (SD) of normal-to-normal (NN) intervals (SDNN), proportion of adjacent NN intervals differing more than 50 ms (pNN50), and root mean square of successive differences between adjacent NN intervals (RMSSD). The closest available sinus rhythm ECG to the CRM date was used to assess PR interval, P wave duration, QRS duration, and QTc interval.

Clinical data were retrieved from electronic medical records, including sex, CHD complexity classification, body mass index (BMI), history of atrial tachyarrhythmia (AT), antiarrhythmic drug (AAD) use, permanent pacemaker (PM) implantation, and transthoracic echocardiography closest to the Holter. LA dilatation was derived from the diagnostic conclusion of the original echocardiographic reports and analyzed as a clinically reported categorical variable. Atrial flutter, intra-atrial reentry tachycardia, and ectopic atrial tachycardia were collectively classified as AT, consistent with the previous study [15]. CHD complexity was stratified as mild, moderate, or severe according to the 2020 European Society of Cardiology guidelines for CHD [16].

### 2.3. Statistical Analysis

The primary endpoint was new-onset AF. The secondary endpoint was AF progression, which was defined as a change from PAF to PeAF, according to the European Society of Cardiology guidelines of AF classification [17]. Based on AF status, patients were categorized into three groups: No-AF, PAF, and PeAF.

Continuous data with normal distribution are reported as mean ± SD and compared using an ANOVA test. Non-normally distributed data are presented as median (interquartile) and analyzed with the Kruskal–Wallis test. Categorical variables are summarized as counts (%) and assessed with chi-square or Fisher’s exact tests, as appropriate. Bonferroni correction was performed to correct for multiple pairwise comparisons. Among patients with new-onset AF, Spearman’s correlation analysis was performed to evaluate whether the interval from baseline CRM to AF diagnosis was associated with baseline ECG- and CRM-derived parameters.

Time-to-event analyses were performed to evaluate risk factors for new-onset AF and progression. AT/PM history at baseline was defined as documented AT or PM implantation before baseline assessment. De novo AT/PM was defined as newly documented AT or PM implantation during follow-up before AF onset or PeAF development and was analyzed as a time-dependent covariate. Cumulative incidence curves were constructed using the Kaplan–Meier method. Univariable Cox proportional hazards regression was used to evaluate potential factors, expressed as hazard ratios (HRs) with 95% confidence intervals (CIs). Variables with *p* < 0.05 in univariable analyses were entered into multivariable Cox regression models with the stepwise method. Sensitivity analyses were performed by repeating the multivariable Cox regression models after excluding patients who developed AF within 1 year and 3 years after the baseline.

Additionally, to account for the competing risk of death, Fine–Gray subdistribution hazard models were additionally applied when evaluating the associations between covariates and the risk of new-onset AF and AF progression. To explore whether the association between AE burden and AF outcomes differed according to LA size, interaction analyses were performed by incorporating multiplicative interaction terms between AE burden and LA dilatation into the multivariable Cox regression models. Ordinal logistic regression was used as a complementary analysis to identify these factors associated with increasing AF severity across ordered AF categories: No-AF, PAF, and PeAF.

To enhance clinical interpretability, continuous variables were rescaled before model fitting: age, SDNN, pNN50, RMSSD, PR interval, QRS duration, and QTc interval were divided by 10 to reflect the effect per 10-unit increase; the number of AE and VE was log-transformed due to skewed distributions. In an additional analysis, AE and VE counts were dichotomized using Youden-derived cut-off values and included in the regression analyses.

For covariates with missing data (detailed in Table S1), we used multiple imputation according to the Markov Chain Monte Carlo method. Five imputed datasets were generated and analyzed separately. Regression estimates were pooled using Rubin’s rules and presented. Bootstrap internal validation of the final multivariable Cox regression model was performed across the five imputed datasets using 1000 resamples per dataset. Harrell’s C-index was calculated from the Cox linear predictor. The optimism-corrected C-index was averaged across imputations, and the 95% confidence interval was derived from the pooled bootstrap distribution. All statistical analyses were conducted using R software (version 4.4.2) and Python (version 3.11). A two-sided *p* < 0.05 was defined as statistical significance.

## 3. Results

### 3.1. Baseline Clinical Characteristics

Among the 573 patients included, cardiac rhythm at the end of the mean follow-up period of 13 ± 5 years was used to categorize patients into the No-AF group (*n* = 466), PAF group (*n* = 72), and PeAF group (*n* = 35). Baseline characteristics at the time of the first CRM of the three groups are presented in Table 1. There were no significant differences in sex, BMI, CHD complexity, presenting symptoms (palpitations, dizziness, syncope), aortic valve or mitral valve dysfunction, or prior surgical repair.

Patients with PeAF were significantly older than those without AF or PAF (No-AF: 33 ± 11 years; PAF: 39 ± 12 years; PeAF: 48 ± 14 years; *p* < 0.001). Compared with the No-AF group, patients with AF had a higher prevalence of LA dilatation (No-AF: 7.9%; PAF: 42.5%; PeAF: 62.5%; *p* < 0.001) and a history of AT (No-AF: 2.6%; PAF: 29.2%; PeAF: 28.6%; *p* < 0.001). Additionally, patients with PAF had a higher rate of PM implantation compared with those without AF (No-AF: 4.3%; PAF: 12.5%; *p* = 0.030). Patients with PeAF had a higher prevalence of moderate-to-severe left ventricular dysfunction compared with the No-AF group (No-AF: 4.3%; PeAF: 24.1%; *p* < 0.001).

### 3.2. Progression of AF

**Figure 1:**
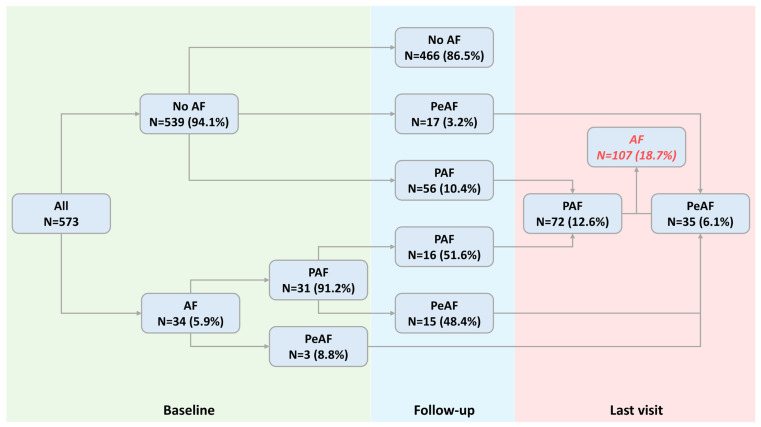
Development and progression of AF during follow-up. The flow diagram illustrates AF status at baseline and transitions between AF subtypes during follow-up. During a mean follow-up of 13 ± 5 years, AF was observed in 107 of 573 patients (18.7%). Among these, 3 patients (2.8%) already had PeAF at baseline, and 32 (29.9%) progressed to PeAF during follow-up. Abbreviations as in Table 1.

As shown in Figure 1, during a mean follow-up of 13 ± 5 years, among the 539 patients (94.1%) without AF at baseline, 73 (13.5%) developed AF within 6 years (range: 0–18 years). Despite the use of AADs in 64 patients (class I: *n* = 1, class II: *n* = 60, class III: *n* = 15, class IV: *n* = 2), 17 patients (23.3%) progressed from PAF to PeAF, with a median progression time of 1 year (range: 0–7 years).

At baseline, 34 (5.9%) CHD patients had already AF, including 31 patients with PAF and 3 patients with PeAF. Among the PAF patients, AADs were used by 28 patients (class I: *n* = 2, class II: *n* = 28, class III: *n* = 10), 15 (48.4%) progressed to PeAF, with a longer median progression time than those who developed AF during follow-up (11 years (range: 3–23 years); *p* < 0.001). However, the age at PeAF diagnosis was comparable between patients with AF at baseline and those with new-onset AF during follow-up (55 ± 12 years vs. 53 ± 16 years, *p* = 0.676).

Overall, 32 (29.9%) out of 107 PAF patients progressed to PeAF during follow-up, with a median progression time of 4 years (range: 0–23 years). Among these 32 patients, progression occurred through different pathways. Thirteen patients (40.6%) progressed from paroxysmal to persistent AF without further transition during follow-up. In 12 patients (37.5%) with PAF, rhythm control was no longer pursued. These patients were therefore classified as direct progression from paroxysmal to permanent AF. Seven patients (21.9%) initially progressed from paroxysmal to persistent AF and subsequently to permanent AF after a median of 1 year (range: 0–14 years), of which AADs were used by all 7 patients (class I: *n* = 1, class II: *n* = 7, class III: *n* = 2).

### 3.3. Baseline ECG and CRM Characteristics

**Figure 2:**
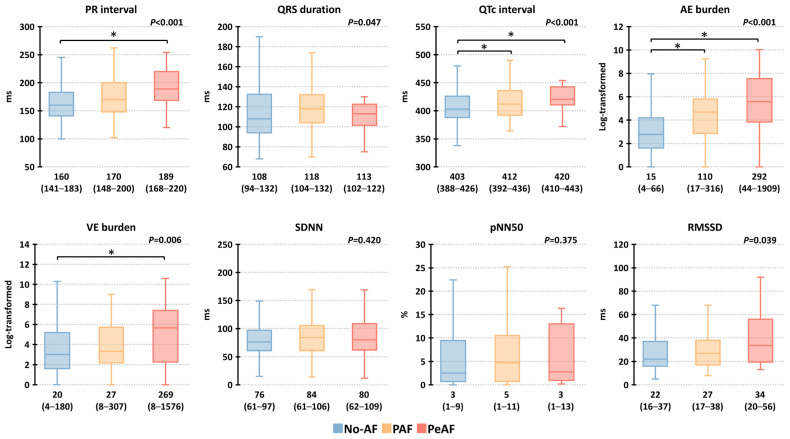
Baseline ECG- and CRM-derived parameters across AF subgroups. AE and VE counts are shown after logarithmic transformation. *p* values indicate overall group differences based on Kruskal–Wallis tests. * indicates significance after Bonferroni correction. AE = atrial ectopy; pNN50 = proportion of successive NN intervals differing by >50 ms; RMSSD = root mean square of successive differences between adjacent NN intervals; SDNN = standard deviation of NN intervals; VE = ventricular ectopy; Other abbreviations as in Table 1.

Baseline ECG and CRM parameters are presented in Figure 2. The PR interval was significantly longer in the PeAF group compared with the No-AF group (No-AF: 160 ms (141–183), PeAF: 189 ms (168–220); *p* = 0.001) at baseline. P-wave duration was also significantly prolonged in both the PAF and PeAF groups compared with the No-AF group (No-AF: 105 ms (100–105), PAF: 109 ms (103–115), PeAF: 115 ms (110–120); *p* < 0.001). In addition, P-wave duration showed a strong positive correlation with PR interval (Spearman’s rho = 0.705, *p* < 0.001).

Similarly, QTc intervals were significantly prolonged in both the PAF and PeAF groups compared with the No-AF group (No-AF: 403 ms (388–426), PAF: 412 ms (392–436), PeAF: 420 ms (410–443); *p* < 0.001). Although a statistically significant difference in QRS duration was observed across the three groups (*p* = 0.047), no significant differences were identified in pairwise comparisons after Bonferroni correction.

Moreover, the total number of AE was significantly higher in both the PAF and PeAF groups compared with the No-AF group (No-AF: 15 (4–66), PAF: 110 (17–316), PeAF: 292 (44–1909); *p* < 0.001). Likewise, VE burden was significantly higher in the PeAF group compared to the No-AF group (No-AF: 20 (4–180), PeAF: 269 (8–1576); *p* = 0.002). However, heart rate variability parameters, including SDNN (No-AF: 76 ms (61–97), PAF: 84 ms (61–106), PeAF: 80 ms (62–109); *p* = 0.420) and pNN50 (No-AF: 3% (1–9), PAF: 5% (1–11), PeAF: 3% (1–13); *p* = 0.375) did not differ among the three groups. Similarly, although RMSSD differed across groups (*p* = 0.039), this difference was not significant after Bonferroni-adjusted pairwise comparisons.

### 3.4. Risk Factors of New-Onset AF and PeAF

**Figure 3:**
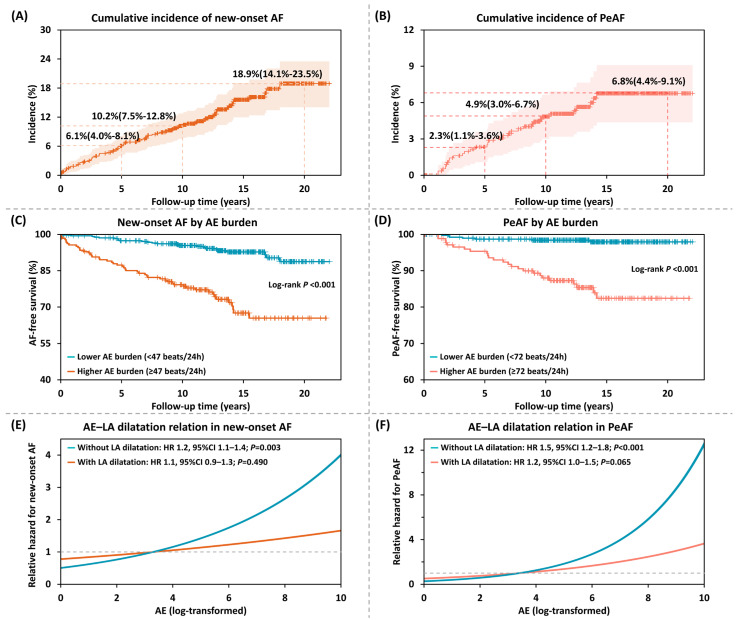
Cumulative incidence of AF, prognostic value of AE burden, and relation between AE and LA dilatation. (**A**) Cumulative incidence of new-onset AF during follow-up. (**B**) Cumulative incidence of PeAF. (**C**) Kaplan–Meier curves for AF-free survival according to the Youden-derived AE burden cut-off of 47 beats/24 h. (**D**) Kaplan–Meier curves for PeAF-free survival according to the Youden-derived AE burden cut-off of 72 beats/24 h. (**E**) Association between AE burden and risk of new-onset AF, stratified by the presence or absence of LA dilatation. AE was significantly associated with new-onset AF in patients without LA dilatation, whereas the association was attenuated in those with LA dilatation. (**F**) Association between AE and risk of PeAF, stratified by LA dilatation status, showing a stronger association in patients without LA dilatation. CI = confidence intervals; HR = hazard ratio; Other abbreviations as in Table 1.

As shown in Figure 3A, the cumulative incidence of new-onset AF was 6.1% (95% CI 4.0–8.1%), 10.2% (7.5–12.8%), and 18.9% (14.1–23.5%) at 5, 10, and 20 years of follow-up, respectively. In univariate Cox regression analyses (Table 2), age, LA dilatation, AE burden, VE burden, PR interval, QTc interval, AT history, and PM history were significantly associated with new-onset AF. After multivariable adjustment, older age (HR per 10 years 1.4, 95% CI 1.2–1.8; *p* = 0.001), LA dilatation (HR 2.6, 95% CI 1.3–5.1; *p* = 0.008), and more AE episodes (HR 1.2, 95% CI 1.1–1.3; *p* = 0.003) remained independently associated with new-onset AF. In addition, both pre-existing AT (HR 4.9, 95% CI 2.5–9.4; *p* < 0.001) and AT developing during follow-up (HR 2.9, 95% CI 1.6–5.2; *p* < 0.001), as well as a history of PM implantation (HR 2.8, 95% CI 1.4–6.0; *p* = 0.006), were strong independent risk factors for AF onset.

Using the Youden index, the optimal cut-off value for AF onset was 47 beats/24 h for AE burden (sensitivity: 67.1%; specificity: 70.8%). Kaplan–Meier analysis showed lower AF-free survival in patients with AE burden ≥47 beats/24 h (log-rank *p* < 0.001, in Figure 3C), and AE burden ≥47 beats/24 h was associated with a 2.8-fold higher risk of new AF onset (95% CI: 1.7–4.7; *p* < 0.001, Table S2). AE burden showed a weak inverse correlation with the interval from baseline CRM to AF diagnosis (Spearman’s r = −0.3, *p* = 0.007, Table S3), and sensitivity analyses excluding AF onset within 1 and 3 years after baseline CRM showed directionally consistent results. Only the AE burden became borderline significant after the 3-year exclusion window (*p* = 0.052, Table S4).

As illustrated in Figure 3B, the cumulative incidence of PeAF was 2.3% (95% CI 1.1–3.6%) at 5 years, 4.9% (3.0–6.7%) at 10 years, and 6.8% (4.4–9.1%) at 20 years. Univariable analyses (Table 3) demonstrated significant associations between PeAF and age, BMI, LA dilatation, AE- and VE burden, RMSSD, PR interval, QTc interval, AT history, and PM implantation. In multivariable Cox models, older age (HR per 10 years 1.8, 95% CI 1.4–2.4; *p* < 0.001), LA dilatation (HR 3.0, 95% CI 1.2–7.0; *p* = 0.014), frequent AE episodes (HR 1.3, 95% CI 1.1–1.5; *p* < 0.001), and a longer PR interval (HR 1.1, 95% CI 1.0–1.2; *p* = 0.028) remained independently associated with the development of PeAF. In bootstrap internal validation, the multivariable Cox model showed a pooled apparent C-index of 0.89 and an optimism-corrected C-index of 0.87, with a bootstrap-derived 95% confidence interval of 0.82–0.93.

Similarly, the optimal cut-off value for PeAF was 72 beats/24 h for AE burden, with a sensitivity of 78.1% and a specificity of 71.7%. Kaplan–Meier analysis also showed lower PeAF-free survival in patients with AE burden ≥72 beats/24 h (log-rank *p* < 0.001, in Figure 3D). An AE burden ≥72 beats/24 h was associated with a 4.7-fold higher risk of PeAF development (95% CI: 1.9–11.4; *p* < 0.001, Table S5).

During follow-up, 31 patients without AF at baseline (6.7%) died before AF onset, and 10 patients with PAF (13.9%) died before PeAF development. Similar results were observed in Fine–Gray regression accounting for the competing risk of death. The main predictors identified in the Cox models remained associated with new-onset AF and PeAF development in the Fine–Gray models (Tables S6 and S7). An ordinal logistic regression analysis was performed with AF categorized as no AF, PAF, and PeAF (Table S8). Similar to the time-to-event analyses, older age, prior surgical repair, LA dilatation, frequent AE episodes, longer PR interval, coexistence of AT, and a history of PM implantation were independently associated with AF persistence.

### 3.5. Interaction Between AE Burden and LA Dilatation

As shown in Figure 3E, although the interaction analysis between AE burden and LA dilatation for new-onset AF was not significant (interaction HR 0.88, 95% CI 0.7–1.2; *p* = 0.378), AE burden was associated with an increased risk of new-onset AF in patients without LA dilatation (HR 1.2, 95% CI 1.1–1.4; *p* = 0.003). In contrast, this association was decreased and not significant among patients with LA dilatation (HR 1.1, 95% CI 0.9–1.3; *p* = 0.490).

Similarly, for PeAF (Figure 3F), the interaction analysis between AE burden and LA dilatation did not reach significance (interaction HR 0.83, 95% CI 0.6–1.1; *p* = 0.250). Nonetheless, a higher AE burden remained strongly associated with PeAF among patients without LA dilatation (HR 1.5, 95% CI 1.2–1.8; *p* < 0.001), whereas the association was weaker and not significant in patients with LA dilatation (HR 1.2, 95% CI 1.0–1.5; *p* = 0.065).

## 4. Discussion

### 4.1. Key Findings

In adults with CHD, prolonged PR and QTc intervals, as well as higher AE and VE burdens, were more frequently observed in patients with AF. PR prolongation was independently associated with AF progression. The AE burden was a strong and independent predictor of both AF onset and progression. The prognostic impact of the AE burden was more pronounced in patients without LA dilatation. In contrast, LA dilatation was a key determinant of AF progression.

### 4.2. Cardiac Ectopy in Relation to AF Onset and Progression

The AE burden in our population is not only an independent predictor of new-onset AF but also AF progression in adults with CHD. A possible explanation is that more AE episodes increase the risk of trigger-driven AF, which is well established in the general population [18,19,20], and this mechanism applies to the CHD population as well. However, unlike the general population in whom ectopic foci primarily originate from the pulmonary veins, CHD patients frequently show non–pulmonary vein triggers, such as superior vena cava, crista terminalis, and coronary sinus [21,22,23]. These atypical trigger locations complicate AF ablation strategies and pulmonary vein isolation alone may therefore be insufficient in the CHD population.

In addition, AE may not only serve as a trigger but may also be an indicator of atrial remodeling. Prior studies have shown that more frequent AE episodes may induce structural and electrophysiological remodeling [24,25]. High-resolution epicardial mapping studies in both general and CHD cohorts have further demonstrated that AE are associated with arrhythmogenic changes, including lower unipolar voltages, a higher prevalence of fractionated potential, and more conduction disorders [26,27]. These findings support the concept that “AE begets AF,” whereby frequent ectopy both initiates and sustains the arrhythmic substrate. Consequently, patients with more AE episodes are not only more prone to develop AF but also more likely to progress into persistent forms of the arrhythmia.

Moreover, although the interaction analysis between AE burden and LA dilatation was not significant, stratified analyses suggested a stronger association between AE burden and AF risk in patients without LA dilatation. This finding suggests that ectopic activity may play a more important role in earlier disease stages, when the atrial structure is relatively preserved. In contrast, once LA dilatation is present, AF risk may be largely driven by structural remodeling, reducing the additional prognostic value of ectopy burden.

Similar to AE, in the current CHD population, a higher VE burden was associated with both new-onset AF and AF progression in univariable analyses, although this association lost significance in multivariable analyses. This finding is in line with previous studies in the general population showing that VEs are associated with an increased risk of incident AF [28,29,30]. One possible explanation is that frequent VE episodes may induce retrograde atrial activation, thereby increasing the risk of AF initiation [31]. Furthermore, frequent VE episodes may lead to impaired left ventricular function, increased LA pressure and stretch, and ultimately more advanced LA remodeling [32,33], which may contribute to both AF onset and progression.

### 4.3. Conduction Intervals and AF Progression

The PR interval reflects intra-atrial and atrioventricular conduction, representing the time needed for an electrical impulse to travel from the sinus node through the atrioventricular node to the His–Purkinje system. In the general population, a prolonged PR interval has been consistently associated with an increased risk of AF onset [14,34,35,36]. Similarly, in our CHD cohort, PR interval was associated with new-onset AF in univariate analyses, though the association diminished after adjusting for confounders. However, PR prolongation remained an independent predictor of AF progression. Multiple studies indicate that prolonged PR intervals are associated with myocardial fibrosis, aging-related remodeling, and impaired conduction properties, which are key factors of AF onset and progression [37,38]. In the present study, P-wave duration was prolonged in patients with PAF and PeAF and showed a strong positive correlation with PR interval. Therefore, while PR prolongation is often a marker of atrioventricular nodal delay, in the CHD population, it may also result from impaired internodal or intra-atrial conduction, owing to structural and surgical alterations [39,40]. Our findings suggest that PR prolongation is not only a marker of atrial structural and electrical remodeling in the general population but also a strong indicator of progressive substrate remodeling in adults with CHD.

The QTc interval, representing the duration of ventricular depolarization and repolarization, is often prolonged in CHD patients, particularly in those with chronic volume overload. Repolarization abnormalities in this population may further be exacerbated by anatomical distortion and post-surgical remodeling [41]. Studies in the general population have shown that QTc prolongation is associated with increased AF susceptibility [12,13]. Both clinical and animal models of congenital long QT syndrome have demonstrated that prolonged atrial action potential durations resulted in an atrial form of “torsades de pointes” and increased susceptibility to AF [42,43]. In our cohort, QTc prolongation was associated with both new-onset AF and AF progression in univariable analyses. Although this association lost statistical significance after adjustment for confounders, prolonged QTc may still represent an important marker of arrhythmic vulnerability in the CHD population and therefore warrants clinical attention.

### 4.4. Coexistence of AT and PM Implantation

Consistent with prior studies, traditional risk factors like advancing age and atrial enlargement emerged as important drivers of AF in CHD. Yet, the high prevalence of AT in CHD adds a layer of complexity. In our cohort, a prior history of AT was independently associated with increased AF risk. In theory, AT can trigger AF and provoke electrical remodeling that predisposes patients to AF. Notably, a recent study suggests that timely ablation of AT may mitigate subsequent AF development in CHD, underscoring the interdependent progression of these arrhythmias [44].

Furthermore, pacing was independently associated with an increased risk of AF onset in the current CHD population. Among the 32 patients with a history of PM implantation, 16 (50%) initially underwent VVI pacing, whereas the remainder received AAI or DDD pacing. VVI pacing, particularly with a right ventricular apical lead, induces atrioventricular and intraventricular asynchrony, leading to adverse remodeling, resulting in left ventricular dysfunction and LA enlargement [45], which promotes AF onset. Moreover, DDD pacing maintains atrioventricular synchrony, but it may still impose mechanical asynchrony when delivered from the right ventricular apex. Hence, minimizing asynchrony ventricular pacing may reduce AF incidence [46,47].

Conduction system pacing, achieved through His bundle pacing or left bundle branch area pacing, directly engages the His–Purkinje system and preserves (or restores) physiological ventricular activation. This approach has been shown to be feasible and safe in patients with CHD [48,49]. Whether conduction system pacing can attenuate atrial remodeling and slow the development of AF in CHD populations remains to be determined and warrants investigation in future prospective studies.

### 4.5. Clinical Implications

Given the increased risk and younger age of AF onset in CHD, early identification of high-risk individuals is crucial. Our results suggest that routine CRM and ECG parameters could be applied during follow-up to stratify risk and personalize surveillance. CHD patients with a high ectopy burden or conduction delay may benefit from intensified rhythm monitoring (prolonged ambulatory ECG, implantable loop recorders, etc.) to facilitate early AF detection and timely rhythm control. Furthermore, in patients with paroxysmal AF, identifying CHD patients at high risk of progression (older age, PR or QTc prolongation, a high ectopy burden, etc.) could prompt early initiation of antiarrhythmic therapy before the arrhythmia becomes persistent. Such a proactive, substrate-oriented strategy may improve rhythm outcomes and reduce the long-term burden of AF in adult CHD care.

### 4.6. Study Limitations

First, due to its retrospective observational design, causality cannot be inferred. Second, ECG and CRM parameters were assessed at a single baseline timepoint. AF onset and progression are dynamic processes, and baseline values may not reflect later electrophysiological changes during follow-up. Although repeat ECG/CRM recordings were available in some patients, they were not collected at standardized time intervals and were often clinically driven, making a reliable trajectory analysis not possible. Therefore, we could not evaluate whether dynamic changes before AF onset provide additional predictive value. Third, LA dilatation was analyzed as a binary variable because continuous LA diameter and LA volume index were not consistently available in this retrospective cohort. This may have reduced statistical power for interaction analyses, and future studies with systematic LA measurements are needed. Higher AE burden was weakly associated with a shorter interval to AF onset, raising the possibility that AE burden partly reflected preclinical electrical changes. However, sensitivity analyses excluding early AF events showed consistent findings, suggesting that the association was not solely driven by recordings obtained shortly before AF diagnosis. Progression to PeAF may have been influenced by variation in rhythm control strategies. While undetected episodes between visits are possible, our focus on persistent/permanent AF reduces the likelihood that transient, asymptomatic episodes significantly affected classification. Death during follow-up may also have influenced the observed risk of AF onset and progression. However, Fine–Gray competing risk analyses with death as a competing event showed results largely consistent with the primary Cox models, supporting the robustness of our findings. The relatively small number of patients with PeAF may result in limited statistical power, unstable estimates, and potential overfitting. Although bootstrap internal validation was performed and suggested good internal discrimination, the C-index should be interpreted cautiously and requires external validation. Studies with larger sample sizes and more variation in atrial size assessed as a continuous variable are needed to further evaluate the role of AE/VE in the initiation and progression of AF.

## 5. Conclusions

In adult patients with CHD, prolonged PR and QTc intervals and frequent AE and VE were more frequently observed in patients with AF. PR prolongation was independently associated with AF progression. Atrial ectopic activity independently predicts AF onset and progression, particularly in CHD patients without LA dilation. In contrast, LA dilatation was a key determinant of AF progression. Noninvasive electrical markers may improve risk stratification and guide early, personalized rhythm management.

## Figures and Tables

**Table 1:** Study population characteristics.

Variables	No AF	PAF	PeAF	*p* Value
N	466	72	35	
Age	33 ± 11 ^†^	39 ± 12 ^†^	48 ± 14 ^†^	<0.001
AF onset age	/	45 ± 13	47 ± 14	0.338
Male	222(47.6%)	41(56.9%)	20(57.1%)	0.217
BMI	24 ± 4	25 ± 4	26 ± 5	0.073
Complexity				0.238
Mild	192(41.2%)	26(36.1%)	15(42.9%)	
Moderate	165(35.4%)	22(30.6%)	15(42.9%)	
Severe	109(23.4%)	24(4.2%)	5(14.3%)	
Symptoms				
Palpitations	164(35.2%)	30(41.7%)	15(42.9%)	0.410
Dizziness	45(9.7%)	4(5.6%)	3(8.6%)	0.527
Syncope	20(4.3%)	4(5.6%)	3(8.6%)	0.482
Surgical repair	418(89.7%)	69(95.8%)	34(97.1%)	0.101
AAD	57(12.2%)	15(20.8%)	9(25.7%)	0.019
AT history	12(2.6%) ^†‡^	21(29.2%) ^†^	10(28.6%) ^‡^	<0.001
PM history	20(4.3%) ^†^	9(12.5%) ^†^	3(8.6%)	0.030
LA dilatation	26(7.9%) ^†‡^	17(42.5%) ^†^	15(62.5%) ^‡^	<0.001
AoV dysfunction	171(44.8%)	32(55.2%)	14(46.7%)	0.333
MV dysfunction	207(55.1%)	41(68.3%)	17(56.7%)	0.156
Left ventricular function				<0.001
Normal function	301(71.7%)	40(63.5%)	18(62.1%)	
Mild dysfunction	101(24.0%)	16(25.4%)	4(13.8%)	
Moderate/severe dysfunction	18(4.3%) ^†^	7(11.4%)	7(24.1%) ^†^	

Values are presented as N (%) or mean ± standard deviation. ^†^ or ^‡^ indicates significance after Bonferroni correction in pairwise comparison. AADs, antiarrhythmic drugs; AF, atrial fibrillation; AoV, aortic valve; AT, atrial tachycardia; BMI, body mass index; LA, left atrium; MV, mitral valve; PAF, paroxysmal AF; PeAF, persistent or permanent AF; PM, permanent pacemaker.

**Table 2:** Univariate and multivariate Cox regression models for new AF onset.

Variables	Univariate			Multivariate		
	HR	95% CI	*p*	HR	95% CI	*p*
Age	1.8	1.5–2.1	<0.001	1.4	1.2–1.8	0.001
Male	1.4	0.9–2.2	0.167			
Surgical repair	2.3	0.7–7.4	0.149			
Severe complexity	1.4	0.9–2.3	0.177			
BMI	1.0	1.0–1.1	0.541			
Moderate/severe MV disease	1.3	0.8–2.1	0.363			
Moderate/severe AoV disease	1.2	0.7–2.0	0.473			
LA dilatation	5.1	3.0–8.8	<0.001	2.6	1.3–5.1	0.008
Impaired left ventricular function	1.2	0.7–2.2	0.468			
AE count	1.3	1.2–1.5	<0.001	1.2	1.1–1.3	0.003
VE count	1.1	1.0–1.2	0.017			
SDNN	1.0	1.0–1.1	0.494			
pNN50	1.1	0.9–1.3	0.496			
RMSSD	1.1	1.0–1.1	0.095			
PR interval	1.1	1.0–1.1	0.003			
QRS duration	1.0	1.0–1.1	0.424			
QTc interval	1.1	1.0–1.2	0.008			
AT						
No AT	Ref					
AT history at baseline	11.1	6.2–19.7	<0.001	4.9	2.5–9.4	<0.001
AT de novo	4.0	2.3–7.0	<0.001	2.9	1.6–5.2	<0.001
PM						
No PM	Ref					
PM history at baseline	3.5	1.7–7.1	0.001	2.8	1.4–6.0	0.006
PM de novo	4.4	2.4–8.1	<0.001	1.8	0.9–3.6	0.095

Age, SDNN, pNN50, RMSSD, PR interval, QRS duration, and QTc interval were scaled per 10 units. AE and VE counts were log-transformed. Abbreviations as in Table 1 and Figure 2.

**Table 3:** Univariate and multivariate Cox regression models for PeAF onset.

Variables	Univariate			Multivariate		
	HR	95% CI	*p*	HR	95% CI	*p*
Age	2.4	1.8–3.1	<0.001	1.8	1.4–2.4	<0.001
Male	1.2	0.6–2.3	0.669			
Surgical repair	3.1	0.4–22.4	0.272			
Severe complexity	0.5	0.2–1.4	0.216			
BMI	1.1	1.0–1.2	0.014			
Moderate/severe MV disease	1.0	0.4–2.1	0.913			
Moderate/severe AoV disease	0.9	0.4–1.8	0.756			
LA dilatation	7.1	3.3–15.5	<0.001	3.0	1.2–7.0	0.014
Impaired left ventricular function	1.4	0.7–3.0	0.333			
AE count	1.5	1.3–1.7	<0.001	1.3	1.1–1.5	<0.001
VE count	1.3	1.1–1.4	0.001			
SDNN	1.0	0.9–1.1	0.835			
pNN50	1.2	1.0–1.6	0.093			
RMSSD	1.1	1.0–1.1	0.046			
PR interval	1.1	1.0–1.2	<0.001	1.1	1.0–1.2	0.028
QRS duration	1.0	0.9–1.2	0.554			
QTc interval	1.1	1.0–1.3	0.011			
AT						
No AT	Ref					
AT history at baseline	7.4	3.4–16.1	<0.001			
AT de novo	2.2	0.8–5.9	0.128			
PM						
No PM	Ref					
PM history at baseline	2.0	0.6–6.7	0.251			
PM de novo	2.6	1.0–6.9	0.049			

Age, SDNN, pNN50, RMSSD, PR interval, QRS duration, and QTc interval were scaled per 10 units. AE and VE counts were log-transformed. Abbreviations as in Table 1 and Figure 2.

## Data Availability

The data underlying this article will be shared on reasonable request to the corresponding author.

## Supplementary Materials

The following supporting information can be downloaded at: https://www.mdpi.com/article/10.3390/jcdd13070293/s1, Table S1: Missing Data Proportions of Covariates Included in the Imputation Model; Table S2. Univariate and multivariate Cox regression models for new AF onset; Table S3. Correlation between time from baseline CRM to new-onset AF and baseline ECG/CRM-derived parameters; Table S4. Multivariable Cox regression for new-onset AF after excluding patients with AF onset within 1 or 3 years after baseline CRM; Table S5. Univariate and multivariate Cox regression models for PeAF onset; Table S6. Subdistribution Hazard Ratios from Fine–Gray Models for new AF onset; Table S7. Subdistribution Hazard Ratios from Fine–Gray Models for PeAF; Table S8. Ordinal Logistic Regression Across AF Types: Univariate and Multivariate Models.

## Abbreviations

The following abbreviations are used in this manuscript:

AAD	Antiarrhythmic drug
AE	Atrial ectopy
AF	Atrial fibrillation
AoV	Aortic valve
AT	Atrial tachycardia
BMI	Body mass index
CHD	Congenital heart disease
CI	Confidence interval
CRM	Continuous rhythm monitoring
ECG	Electrocardiography
HR	Hazard ratio
LA	Left atrial/left atrium
MV	Mitral valve
NN	Normal-to-normal interval
PAF	Paroxysmal atrial fibrillation
PeAF	Persistent or permanent atrial fibrillation
PM	Permanent pacemaker
pNN50	Proportion of successive NN intervals differing by >50 ms
QTc	Corrected QT interval
RMSSD	Root mean square of successive differences between adjacent NN intervals
SD	Standard deviation
SDNN	Standard deviation of NN intervals
VE	Ventricular ectopy
