# Supplementary material for: From Minor Monitoring to Major Insight: Predicting AF Development in the Congenital Heart Disease Population
Source: J Cardiovasc Dev Dis. 2026 Jun 24;13(7):293. doi: 10.3390/jcdd13070293 (PMC13409886; doi:10.3390/jcdd13070293)
Supplement: Supplementary file 1 [file jcdd-13-00293-s001.zip › jcdd-4342993-supplementary.pdf]

Table S1. Missing Data Proportions of Covariates Included in the Imputation Model

Variables	Missing (rate)
BMI	60(10.5%)
Mitral valve disease	107(18.7%)
Aortic valve disease	103(18.0%)
Left ventricular function	61(10.6%)
LA dilatation	181(31.6%)
SDNN	129(22.5%)
pNN50	129(22.5%)
RMSSD	129(22.5%)
PR interval	25(4.4%)
QRS duration	25(4.4%)
QTC interval	25(4.4%)

Values are presented as N (%).

BMI, body mass index; LA, left atrial; pNN50, proportion of successive NN intervals differing by >50 ms; RMSSD, root mean square of successive differences between adjacent NN intervals; SDNN, standard deviation of NN intervals;

Table S2. Univariate and multivariate Cox regression models for new AF onset.

Variables	Univariate			Multivariate		
	HR	95% CI	*P*	HR	95% CI	*P*
Age	1.8	1.5-2.1	<0.001	1.4	1.2-1.8	0.001
Male	1.4	0.9-2.2	0.167			
Surgical repair	2.3	0.7-7.4	0.149			
Severe complexity	1.4	0.9-2.3	0.177			
BMI	1.0	1.0-1.1	0.541			
Moderate/severe MV disease	1.3	0.8-2.1	0.363			
Moderate/severe AoV disease	1.2	0.7-2.0	0.473			
LA dilatation	5.1	3.0-8.8	<0.001	2.4	1.3-4.7	0.009
Impaired left ventricular function	1.2	0.7-2.2	0.468			
Higher AE burden (⩾47 beats/24h)	4.7	2.9-7.6	<0.001	2.8	1.7-4.7	<0.001
Higher VE burden (⩾88 beats/24h)	1.9	1.2-3.0	0.006			
SDNN	1.0	1.0-1.1	0.494			
pNN50	1.1	0.9-1.3	0.496			
RMSSD	1.1	1.0-1.1	0.095			
PR interval	1.1	1.0-1.1	0.003			
QRS duration	1.0	1.0-1.1	0.424			
QTc interval	1.1	1.0-1.2	0.008			
AT						
No AT	Ref					
AT history at baseline	11.1	6.2-19.7	<0.001	5.2	2.7-9.8	<0.001
AT de novo	4.0	2.3-7.0	<0.001	2.7	1.5-4.8	<0.001
PM						
No PM	Ref					
PM history at baseline	3.5	1.7-7.1	0.001	3.1	1.5-6.4	0.002
PM de novo	4.4	2.4-8.1	<0.001	1.9	0.9-3.6	0.073

Age, SDNN, pNN50, RMSSD, PR interval, QRS duration, and QTc interval were scaled per 10 units. AE and VE counts were categorized based on cut-off values determined by the Youden index.

AE, atrial ectopy; AF, atrial fibrillation; AoV, aortic valve; AT, atrial tachycardia; BMI, body mass index; CI, confidence intervals; HR, hazard ratio; LA, left atrium; MV, mitral valve; PM, permanent pacemaker; pNN50, proportion of successive NN intervals differing by >50ms; RMSSD, root mean square of successive differences between adjacent NN intervals; SDNN, standard deviation of NN intervals; VE, ventricular ectopy.

Table S3. Correlation between time from baseline CRM to new-onset AF and baseline ECG/CRM-derived parameters.

Baseline parameter	Spearman's r	P
AE count	-0.3	**0.007**
VE count	-0.1	0.255
SDNN	0.0	0.947
NN>50	0.0	0.744
RMSSD	-0.1	0.634
PR	0.0	0.972
QRS	0.0	0.803
QTC	-0.1	0.265

AE, atrial ectopy; AF, atrial fibrillation; pNN50, proportion of successive NN intervals differing by >50ms; RMSSD, root mean square of successive differences between adjacent NN intervals; SDNN, standard deviation of NN intervals; VE, ventricular ectopy.

Table S4. Multivariable Cox regression for new-onset AF after excluding patients with AF onset within 1 or 3 years after baseline CRM

	Multivariate Cox regression							
	**HR**	**95% CI**		**P**	**HR**	**95% CI**		**P**
	Excluding AF onset within 1 year				Excluding AF onset within 3 years			
**Age**	1.32	1.06	1.66	**0.015**	1.36	1.06	1.74	**0.014**
**LA dilatation**	2.84	1.34	6.04	**0.007**	2.16	1.00	4.64	**0.049**
**AE count**	1.17	1.05	1.31	**0.005**	1.13	1.00	1.27	0.052
**AT**								
No AT	Ref				Ref			
AT history at baseline	4.60	2.18	9.68	**0.000**	4.77	2.16	10.51	**0.000**
AT de novo	3.08	1.66	5.70	**0.000**	2.93	1.48	5.82	**0.002**
**PM**								
No PM	Ref				Ref			
PM history at baseline	2.47	1.06	5.77	**0.037**	3.53	1.52	8.23	**0.003**
PM de novo	2.28	1.12	4.65	**0.023**	2.88	1.33	6.22	**0.007**

Age was scaled per 10 units. AE counts were log-transformed.

AE, atrial ectopy; AF, atrial fibrillation; AT, atrial tachycardia; CI, confidence intervals; LA, left atrium; PM, permanent pacemaker; HR, hazard ratio.

Table S5. Univariate and multivariate cox regression models for PeAF onset.

Variables	Univariate			Multivariate		
	HR	95% CI	*P*	HR	95% CI	*P*
Age	2.4	1.8-3.1	**<0.001**	1.8	1.4-2.5	**<0.001**
Male	1.2	0.6-2.3	0.669			
Surgical repair	3.1	0.4-22.4	0.272			
Severe complexity	0.5	0.2-1.4	0.216			
BMI	1.1	1.0-1.2	**0.014**			
Moderate/severe MV disease	1.0	0.4-2.1	0.913			
Moderate/severe AoV disease	0.9	0.4-1.8	0.756			
LA dilatation	7.1	3.3-15.5	**<0.001**	2.9	1.3-6.7	**0.012**
Impaired left ventricular function	1.4	0.7-3.0	0.333			
Higher AE burden (≥72 beats/24h)	8.6	3.7-20.0	**<0.001**	4.7	1.9-11.4	**<0.001**
Higher VE burden (≥249 beats/24h)	3.6	1.8-7.3	**<0.001**			
SDNN	1.0	0.9-1.1	0.835			
pNN50	1.2	1.0-1.6	0.093			
RMSSD	1.1	1.0-1.1	**0.046**			
PR interval	1.1	1.0-1.2	**<0.001**	1.1	1.0-1.2	**0.013**
QRS duration	1.0	0.9-1.2	0.554			
QTc interval	1.1	1.0-1.3	**0.011**			
AT						
No AT	Ref					
AT history at baseline	7.4	3.4-16.1	**<0.001**			
AT de novo	2.2	0.8-5.9	0.128			
PM						
No PM	Ref					
PM history at baseline	2.0	0.6-6.7	0.251			
PM de novo	2.6	1.0-6.9	**0.049**			

Age, SDNN, pNN50, RMSSD, PR interval, QRS duration, and QTc interval were scaled per 10 units. AE and VE counts were categorized based on cut-off values determined by the Youden index.

AE, atrial ectopy; AF, atrial fibrillation; AoV, aortic valve; AT, atrial tachycardia; BMI, body mass index; CI, confidence intervals; HR, hazard ratio; LA, left atrium; MV, mitral valve; PM, permanent pacemaker; pNN50, proportion of successive NN intervals differing by >50ms; RMSSD, root mean square of successive differences between adjacent NN intervals; SDNN, standard deviation of NN intervals; VE, ventricular ectopy.

Table S6. Subdistribution Hazard Ratios from Fine–Gray Models for new AF onset.

**Variables**	**Univariate**			**Multivariate**		
	sHR	95% CI	*P*	sHR	95% CI	*P*
Age	1.7	1.4-2.1	**<0.001**	1.3	1.0-1.7	**0.027**
Male	1.4	0.9-2.2	0.170			
Surgical repair	2.4	0.7-7.7	0.140			
Severe complexity	1.2	0.9-1.5	0.240			
BMI	1.0	1.0-1.1	**0.504**			
Moderate/severe MV disease	1.3	0.8-2.1	0.373			
Moderate/severe AoV disease	1.2	0.7-2.0	0.447			
LA dilatation	4.9	2.9-8.4	**<0.001**	2.5	1.1-5.4	**0.022**
Impaired left ventricular function	1.2	0.7-2.1	0.510			
AE count	1.3	1.2-1.5	**<0.001**	1.2	1.0-1.3	**0.017**
VE count	1.1	1.0-1.2	**0.018**			
SDNN	1.0	1.0-1.1	0.456			
pNN50	1.1	0.9-1.3	0.489			
RMSSD	1.1	1.0-1.1	**0.057**			
PR interval	1.1	1.0-1.1	**<0.001**			
QRS duration	1.0	1.0-1.1	0.385			
QTc interval	1.1	1.0-1.2	**0.008**			
AT						
No AT	Ref					
AT history at baseline	10.3	5.8-18.4	**<0.001**	4.5	2.2-9.6	**<0.001**
AT de novo	4.0	2.3-7.0	**<0.001**	2.9	1.5-5.5	**0.001**
PM						
No PM	Ref					
PM history at baseline	3.2	1.6-6.5	0.001	2.5	1.1-5.8	**0.026**
PM de novo	4.2	2.3-7.8	**<0.001**	1.6	0.7-3.7	0.322

Age, SDNN, pNN50, RMSSD, PR interval, QRS duration, and QTc interval were scaled per 10 units. AE and VE counts were log-transformed.

AE, atrial ectopy; AF, atrial fibrillation; AoV, aortic valve; AT, atrial tachycardia; BMI, body mass index; CI, confidence intervals; LA, left atrium; MV, mitral valve; PM, permanent pacemaker; pNN50, proportion of successive NN intervals differing by >50ms; RMSSD, root mean square of successive differences between adjacent NN intervals; SDNN, standard deviation of NN intervals; sHR, subdistribution hazard ratio; VE, ventricular ectopy.

Table S7. Subdistribution Hazard Ratios from Fine–Gray Models for PeAF.

Variables	Univariate			Multivariate		
	sHR	95% CI	*P*	sHR	95% CI	*P*
Age	2.3	1.8-3.0	**<0.001**	1.8	1.3-2.5	**0.001**
Male	1.2	0.6-2.3	0.660			
Surgical repair	3.1	0.4-22.4	0.270			
Severe complexity	0.7	0.4-1.2	0.190			
BMI	1.1	1.0-1.2	**0.009**			
Moderate/severe MV disease	0.9	0.4-2.1	0.892			
Moderate/severe AoV disease	0.9	0.4-1.8	0.758			
LA dilatation	6.9	3.2-15.1	**<0.001**	3.0	1.2-7.1	**0.021**
Impaired left ventricular function	1.4	0.7-2.9	0.346			
AE count	1.5	1.3-1.7	**<0.001**	1.3	1.1-1.6	**0.002**
VE count	1.2	1.1-1.4	**0.003**			
SDNN	1.0	0.9-1.1	0.806			
pNN50	1.2	1.0-1.6	0.085			
RMSSD	1.1	1.0-1.1	**0.012**			
PR interval	1.1	1.0-1.2	**0.001**	1.1	1.0-1.2	**0.044**
QRS duration	1.0	0.9-1.2	0.559			
QTc interval	1.1	1.0-1.3	**0.011**			
AT						
No AT	Ref					
AT history at baseline	7.2	3.3-15.5	**<0.001**			
AT de novo	2.2	0.8-6.0	0.120			
PM						
No PM	Ref					
PM history at baseline	1.9	0.6-6.2	0.280			
PM de novo	2.6	1.0-6.6	**0.042**			

Age, SDNN, pNN50, RMSSD, PR interval, QRS duration, and QTc interval were scaled per 10 units. AE and VE counts were log-transformed.

AE, atrial ectopy; AF, atrial fibrillation; AoV, aortic valve; AT, atrial tachycardia; BMI, body mass index; CI, confidence intervals; LA, left atrium; MV, mitral valve; PeAF, persistent or permanent AF; PM, permanent pacemaker; pNN50, proportion of successive NN intervals differing by >50ms; RMSSD, root mean square of successive differences between adjacent NN intervals; SDNN, standard deviation of NN intervals; sHR, subdistribution hazard ratio; VE, ventricular ectopy.

Table S8. Ordinal Logistic Regression Across AF Types: Univariate and Multivariate Models.

**Variables**	**Univariate**			**Multivariate**		
	OR	95% CI	*P*	OR	95% CI	*P*
Age	2.0	1.6-2.4	**<0.001**	1.7	1.4-2.2	**0.001**
Male	1.5	1.0-2.2	0.083			
Surgical repair	3.0	1.1-8.4	0.041	3.3	1.0-10.4	**0.043**
Severe complexity	1.2	0.7-1.9	0.555			
BMI	1.1	1.0-1.1	**0.029**			
Moderate/severe MV disease	1.4	0.9-2.1	0.188			
Moderate/severe AoV disease	1.2	0.8-1.9	0.399			
LA dilatation	5.9	3.4-10.1	**<0.001**	2.5	1.3-4.8	**0.006**
Impaired left ventricular function	1.6	1.0-2.5	0.078			
AE count	1.4	1.3-1.5	**<0.001**	1.2	1.1-1.4	**<0.001**
VE count	1.1	1.0-1.2	**0.004**			
SDNN	1.1	1.0-1.1	0.175			
pNN50	1.2	1.0-1.4	0.091			
RMSSD	1.1	1.0-1.1	**0.028**			
PR interval	1.1	1.1-1.2	**<0.001**	1.1	1.0-1.2	**0.006**
QRS duration	1.1	1.0-1.1	0.096			
QTc interval	1.1	1.1-1.2	**<0.001**			
AT						
No AT	Ref					
AT history at baseline	14.1	7.6-26.4	**<0.001**	6.398	3.2-12.9	**<0.001**
AT de novo	4.7	2.6-8.5	**<0.001**	4.082	2.1-7.9	**<0.001**
PM						
No PM	Ref					
PM history at baseline	3.1	1.5-6.5	0.002	3.827	1.6-9.0	**0.002**
PM de novo	5.1	2.7-9.7	**<0.001**	2.0	0.9-4.3	0.098

Age, SDNN, pNN50, RMSSD, PR interval, QRS duration, and QTc interval were scaled per 10 units. AE and VE counts were log-transformed.

AE, atrial ectopy; AF, atrial fibrillation; AoV, aortic valve; AT, atrial tachycardia; BMI, body mass index; CI, confidence intervals; LA, left atrium; MV, mitral valve; OR, Odds Ratio; PM, permanent pacemaker; pNN50, proportion of successive NN intervals differing by >50ms; RMSSD, root mean square of successive differences between adjacent NN intervals; SDNN, standard deviation of NN intervals; VE, ventricular ectopy.
